# Persistent Overexpression of Phosphoglycerate Mutase, a Glycolytic Enzyme, Modifies Energy Metabolism and Reduces Stress Resistance of Heart in Mice

**DOI:** 10.1371/journal.pone.0072173

**Published:** 2013-08-12

**Authors:** Junji Okuda, Shinnichiro Niizuma, Tetsuo Shioi, Takao Kato, Yasutaka Inuzuka, Tsuneaki Kawashima, Yodo Tamaki, Akira Kawamoto, Yohei Tanada, Yoshitaka Iwanaga, Michiko Narazaki, Tetsuya Matsuda, Souichi Adachi, Tomoyoshi Soga, Genzou Takemura, Hiroshi Kondoh, Toru Kita, Takeshi Kimura

**Affiliations:** 1 Department of Cardiovascular Medicine, Graduate School of Medicine, Kyoto University, Kyoto, Japan; 2 Department of Systems Science, Graduate School of Informatics, Kyoto University, Kyoto, Japan; 3 Human Health Sciences, Graduate School of Medicine, Kyoto University, Kyoto, Japan; 4 Institute for Advanced Biosciences, Keio University, Tsuruoka, Japan; 5 Division of Cardiology, Gifu University Graduate School of Medicine, Gifu, Japan; 6 Department of Geriatric Medicine, Graduate School of Medicine, Kyoto University, Kyoto, Japan; Rutgers New Jersey Medical School, United States of America

## Abstract

**Background:**

Heart failure is associated with changes in cardiac energy metabolism. Glucose metabolism in particular is thought to be important in the pathogenesis of heart failure. We examined the effects of persistent overexpression of phosphoglycerate mutase 2 (Pgam2), a glycolytic enzyme, on cardiac energy metabolism and function.

**Methods and Results:**

Transgenic mice constitutively overexpressing Pgam2 in a heart-specific manner were generated, and cardiac energy metabolism and function were analyzed. Cardiac function at rest was normal. The uptake of analogs of glucose or fatty acids and the phosphocreatine/βATP ratio at rest were normal. A comprehensive metabolomic analysis revealed an increase in the levels of a few metabolites immediately upstream and downstream of Pgam2 in the glycolytic pathway, whereas the levels of metabolites in the initial few steps of glycolysis and lactate remained unchanged. The levels of metabolites in the tricarboxylic acid (TCA) cycle were altered. The capacity for respiration by isolated mitochondria *in vitro* was decreased, and that for the generation of reactive oxygen species (ROS) *in vitro* was increased. Impaired cardiac function was observed in response to dobutamine. Mice developed systolic dysfunction upon pressure overload.

**Conclusions:**

Constitutive overexpression of Pgam2 modified energy metabolism and reduced stress resistance of heart in mice.

## Introduction

Heart failure is becoming a serious health care problem. It is a typical age-related disease, and the number of patients with heart failure continues to increase [1]. Moreover, it is the most frequent cause of rehospitalization in all cases of disease [2]. However, even with the best treatment, the annual rate of mortality from heart failure is still as high as 10%. Thus, the development of new treatments is a major challenge in cardiology [1]. The development and progression of heart failure is associated with changes in cardiac energy metabolism, including altered substrate utilization, abnormal mitochondrial function, and a decrease in energy transfer due to creatine shuttle dysfunction [3]. Among these changes, modulating substrate utilization appears to be a promising therapeutic target. Partial inhibition of fatty acid utilization, which is likely to increase glucose metabolism, has been shown to ameliorate cardiac dysfunction in patients with heart failure [4]. A previous study showed that a β-adrenergic receptor blocker shifted substrate utilization from fatty acids to glucose [5].

How substrate utilization is altered in patients with heart failure remains controversial. Previous studies reported that fatty acid utilization in patients with heart failure was increased [6], [7] or decreased [8]. Glucose utilization in patients with heart failure has been reported to increase [8], [9] as well as decrease [6], [7]. Fatty acid utilization was shown to be unchanged [10] or decreased [11] in animal models of heart failure, while glucose utilization increased in animals with cardiac hypertrophy [12] or heart failure [11]. We reported previously that the uptake of an analog of fatty acids was decreased and that of glucose was increased in a rat model of heart failure [13].

Cardiac-specific overexpression of glucose transporter 1 (GLUT1) was shown to result in increased glucose uptake, glycolysis, and decreased fatty acid oxidation, and also prevents systolic dysfunction and left ventricular dilatation in mice subjected to pressure overload induced by ascending aortic constriction [14]. Pyruvate dehydrogenase kinase (PDK) inactivates pyruvate dehydrogenase, the rate-limiting enzyme of glycolysis. Cardiac-specific overexpression of pyruvate dehydrogenase kinase 4 (PDK4) in transgenic mice has been shown to decrease glucose oxidation and increase fatty acid catabolism, and predispose animals to heart failure [15]. The potential advantage to increasing glucose utilization is that utilizing glucose as an energy source stoichiometrically requires less oxygen than that of fatty acids to produce the same amount of adenosine triphosphate (ATP) [16].

The phosphoglycerate mutase (Pgam) protein is an important enzyme in the glycolytic pathway and catalyzes the transfer of phosphate groups from 3-phosphoglycerate to 2-phosphoglycerate. This enzyme functions as a dimer and has been highly conserved throughout evolution. Mammals express two isoforms, one of which is brain-specific (Pgam1) while the other is muscle-specific (Pgam2) [17]. The overexpression of Pgam2 using a retroviral vector in primary mouse embryonic fibroblasts (MEFs) was shown to enhance glycolysis [18]. In humans, a deficiency in phosphoglycerate mutase caused glycogen storage disease type X, characterized by exercise intolerance and cramps [19]. Tissue Pgam1 protein levels were increased and associated with poor clinical outcome in patients with lung cancer [20]. Moreover, inhibiting the Pgam1 protein was shown to attenuate tumor growth [21]. A small-molecule inhibitor of the Pgam1 protein has been developed [22].

Heart tissue has the second highest level of Pgam activity, next to skeletal muscle [23]. Pgam2 protein expression increased approximately 5-fold in a canine model of tachycardia-induced heart failure [24]. These results indicate that Pgam2 may be involved in the development of heart failure. To examine the role of Pgam2 in the heart, we generated transgenic mice constitutively overexpressing Pgam2 in a heart-specific manner and analyzed cardiac energy metabolism and function.

## Materials and Methods

### 1. Animals

Transgenic lines overexpressing murine Pgam2 in a heart-specific manner were generated on a C57BL/6J (C57BL6) background using the murine α-myosin heavy chain (α-MHC) promoter [25], and were designated Pgam2 mice. The expression of the α-MHC promoter markedly increases several days after birth and remains high throughout the lifetime of these mice [26]. Transgenic mice and non-transgenic (NTg) littermates as controls were maintained on a 12-h light/dark cycle, fed a normal laboratory diet *ad libitum*, sacrificed by decapitation under ether anesthesia at 3 months of age, and analyzed. Hearts were resected immediately, washed in cold phosphate-buffered saline (PBS), divided into 3 parts, snap-frozen in liquid nitrogen, and stored at -80°C. Ventricular tissues were used for western blotting, enzymatic activity assays, metabolomic analysis, measuring mitochondrial oxygen consumption and hydrogen peroxide (H_2_O_2_) generation, and histological analysis. This investigation conformed to the Guide for the Care and Use of Laboratory Animals published by the US National Institutes of Health (NIH Publication No. 85-23, revised 1996). All animal care, experiments, and methods were approved by the Animal Care and Use Committees of Kyoto University Graduate School of Medicine.

### 2. Western blotting

Total protein was extracted from frozen hearts, resolved, electrophoresed, and electrotransferred to a nitrocellulose membrane as described previously [27]. The antibodies used for western blotting were those against Pgam1 (1∶1000, Abcam, Cambridge, UK; Ab2220), Myc (9E10) (1∶1000; Santa Cruz Biotechnology, Santa Cruz, CA, USA; sc-40), and glyceraldehyde 3-phosphate dehydrogenase (GAPDH) (1∶2000; Chemicon International, Temecula, CA, USA; 6C5).

### 3. Measuring Pgam and phosphofructokinase (PFK) activity

The assay of phosphoglycerate mutase activity, measured via activity-based nicotinamide adenine dinucleotide (NADH)-consuming spectrophotometry, was performed as described [18]. Fifteen μg of tissue lysate was incubated with NADH (0.2 mmol/L; Sigma, St. Louis, MO, USA; N7410), adenosine diphosphate (ADP) (1.5 mmol/L, Sigma, A4386), 2,3-diphosphoglycerate (10 µmol/L, Sigma, D9134), lactate dehydrogenase (200 milliunits, Sigma, L1254), pyruvate kinase (170 milliunits, Sigma, P7768), and enolase (35 milliunits, Sigma, E0379) at 37°C for 10 minutes for the Pgam assay. The reaction was started by adding 3-phosphoglyceric acid (3-PGA) (1 mmol/L final concentration, Sigma, P8877) as a substrate to the assay mixture. The reduced form (NADH) and not the oxidized form (NAD^+^) absorbs ultraviolet rays at 340 nm, and absorbance at 340 nm was previously shown to be proportional to the concentration of NADH; therefore, we monitored the decrease in absorbance of the above reaction mixture at 340 nm under incubation at 37°C over a time period of 12 minutes in a Spectra max M2^e^ plate reader (Molecular Devices, Menlo Park, CA, USA). Phosphofructokinase (PFK) activity was measured using the Phosphofructokinase Activity Colorimetric Assay Kit (BioVision, Milpitas, CA, USA; K776-100), according to the manufacturer's instructions. Briefly, PFK converts fructose-6-phosphate (F6P) and ATP to fructose-diphosphate and ADP. ADP is then converted to adenosine monophosphate (AMP) and NADH in the presence of the substrate and enzyme mix. NADH reduces a colorless probe to a colored product that exhibits strong absorbance at 450 nm. We measured absorbance at 450 nm to analyze the amount of NADH produced, which reflected PFK activity levels.

### 4. Cardiac echocardiography

Cardiac echocardiography was performed as described previously, using an intraperitoneal injection of 2-2-2 tribromoethanol (240 mg/kg, Wako Pure Chemical, Kyoto, Japan) as an anesthetic [27], [28].

### 5. Myocardial uptake of glucose and fatty acids

Myocardial uptake of glucose and fatty acids was analyzed using the analogs ^18^F-fluorodeoxyglucose (^18^FDG) and ^125^I-labeled 15-(*p*-iodophenyl)-9-*R,S*-methylpentadecanoic acid (^125^I-9MPA), respectively, as previously described [13]. The amount of radioisotope incorporated was presented as a standard uptake value (SUV). SUV: tissue concentration (MBq/g)/(injected dose (MBq)/body weight (g)).

### 6. In situ ^31^P magnetic resonance spectroscopy (MRS)

Myocardial energy reserve was measured by *in situ* ^31^P magnetic resonance spectroscopy (MRS) using a Bruker Biospec 70/20 USR system (Bruker Biospin, Ettlingen, Germany) with a 20-mm-diameter ^1^H/^31^P surface coil as described [29]. Isoflurane (2%) was used for anesthesia. The phosphocreatine (PCr)/βATP ratio was calculated by dividing PCr resonance by that of β-ATP and was used as an index of cardiac energy reserve.

### 7. Metabolomic Analysis of Pgam2 mice

The ventricular heart tissue of Pgam2 mice and their NTg littermates at 3 months of age (n = 5 in each group) was subjected to metabolomic analysis as described [13]. Capillary electrophoresis time-of-flight mass spectrometry (CE-TOFMS) was used for metabolomic analysis [30]. We measured the levels of metabolites in glycolysis and the tricarboxylic acid (TCA) cycle, and amino acids and their derivatives.

### 8. Quantitative real-time polymerase chain reaction (PCR)

Preparing RNA and quantitative real-time PCR were performed as described previously [28]. The level of each mRNA was normalized with 18S ribosomal mRNA as an endogenous control. The genes and primer sequences analyzed are listed in Table S1.

### 9. Measurement of oxygen consumption using isolated mitochondria

Mitochondria were isolated from the heart as described previously [31]. Mitochondria obtained by this method were likely to be in the subsarcolemmal fraction [31]. Oxygen consumption by isolated mitochondria was measured using a Clark oxygen electrode cuvette (Yellow Springs Instruments, Yellow Springs, OH, USA), as described [32]. Briefly, substrates and inhibitors corresponding to individual segments of the electron transport pathway were added in turn to samples in the oxygen electrodes in the following final concentrations (5 mM malate + 5 mM pyruvate, 100 nM rotenone, 5 mM succinate, 1 mM adenosine diphosphate, 50 nM antimycin A, 0.4 mM N,N,N',N'-tetramethyl-p-phenylenediamine (TMPD) + 1 mM ascorbate, and 5 mM potassium cyanide).

### 10. Measuring mitochondrial H_2_O_2_ generation using isolated mitochondria

Mitochondria were isolated using a Qproteome Mitochondria Isolation Kit (Qiagen, Valencia, CA, USA), and mitochondrial H_2_O_2_ release was measured in the presence of horseradish peroxidase using an Amplex Red Hydrogen Peroxide/Peroxidase Assay kit (Molecular Probes, Carlsbad, CA, USA) as described [32]. The substrates used were 2.5 mM malate + 2.5 mM pyruvate or 5 mM succinate. Measurements were performed with 96-well plates under incubation at 37°C. Fluorometric measurements were performed with excitation at 544 nm and emission at 590 nm.

### 11. Transmission electron microscopy

Cardiac tissue from the left ventricle was quickly cut into 1 mm cubes, immersion-fixed with 2.5% glutaraldehyde in 0.1 mol/L phosphate buffer (pH 7.4) overnight at 4°C, and fixed in 1% buffered osmium tetroxide. Specimens were subsequently dehydrated through a graded ethanol series and embedded in epoxy resin. Ultrathin slices (90 nm) were double-stained with uranyl acetate and lead citrate, and observed under an electron microscope (H-800, Hitachi, Tokyo, Japan). Morphometrical analyses were performed as described [33].

### 12. Measuring thiobarbituric acid reactive substrates (TBARS)

TBARS levels in left ventricular tissue were measured according to the manufacturer's instructions (Alexis Biochemicals, Lausen, Switzerland).

### 13. Cardiac hemodynamics in vivo assessed by cardiac catheterization under dobutamine infusion

Left ventricular functional reserve was analyzed by cardiac catheterization under dobutamine infusion as reported previously. Isoflurane (2%) was used for anesthesia [28]. LV pressure signals of 10 to 20 beats were averaged and analyzed with PowerLab software (Chart 5, AD Instruments, Dunedin, New Zealand).

### 14. Surgical procedures for transverse aortic constriction (TAC)

Transverse aortic constriction (TAC) or a sham operation was performed on 12-week-old male NTg and Pgam2 mice as described previously [34]. Briefly, a 7-0 silk string ligature was tied around a 26-gauge needle and the needle was then removed to constrict the transverse aorta. Mice were sacrificed and analyzed 14 days after aortic constriction.

### 15. Measuring myocardial fibrosis

Hearts were fixed in 4% paraformaldehyde (PFA), embedded in paraffin, and sectioned (10 µm) for histological evaluation. Sirius Red staining was performed and images were captured and digitized using an image analyzing light microscopy system (KEYENCE BioZero BZ-8000). To quantify the area of myocardial interstitial fibrosis, the red pixel content of the digitized photos was measured relative to the total tissue area using Adobe Photoshop 7.0.1 software (Adobe Systems, San Jose, CA, USA).

### 16. Statistical analysis

Values are expressed as the mean ± SEM for each experimental group. ANOVA was used for comparisons between multiple groups. The Student's *t* test was used for comparisons between 2 groups. In all tests, a value of p<0.05 was considered significant.

## Results

### 1. Generation of transgenic mice constitutively overexpressing Pgam2 in the heart

**Figure 1:**
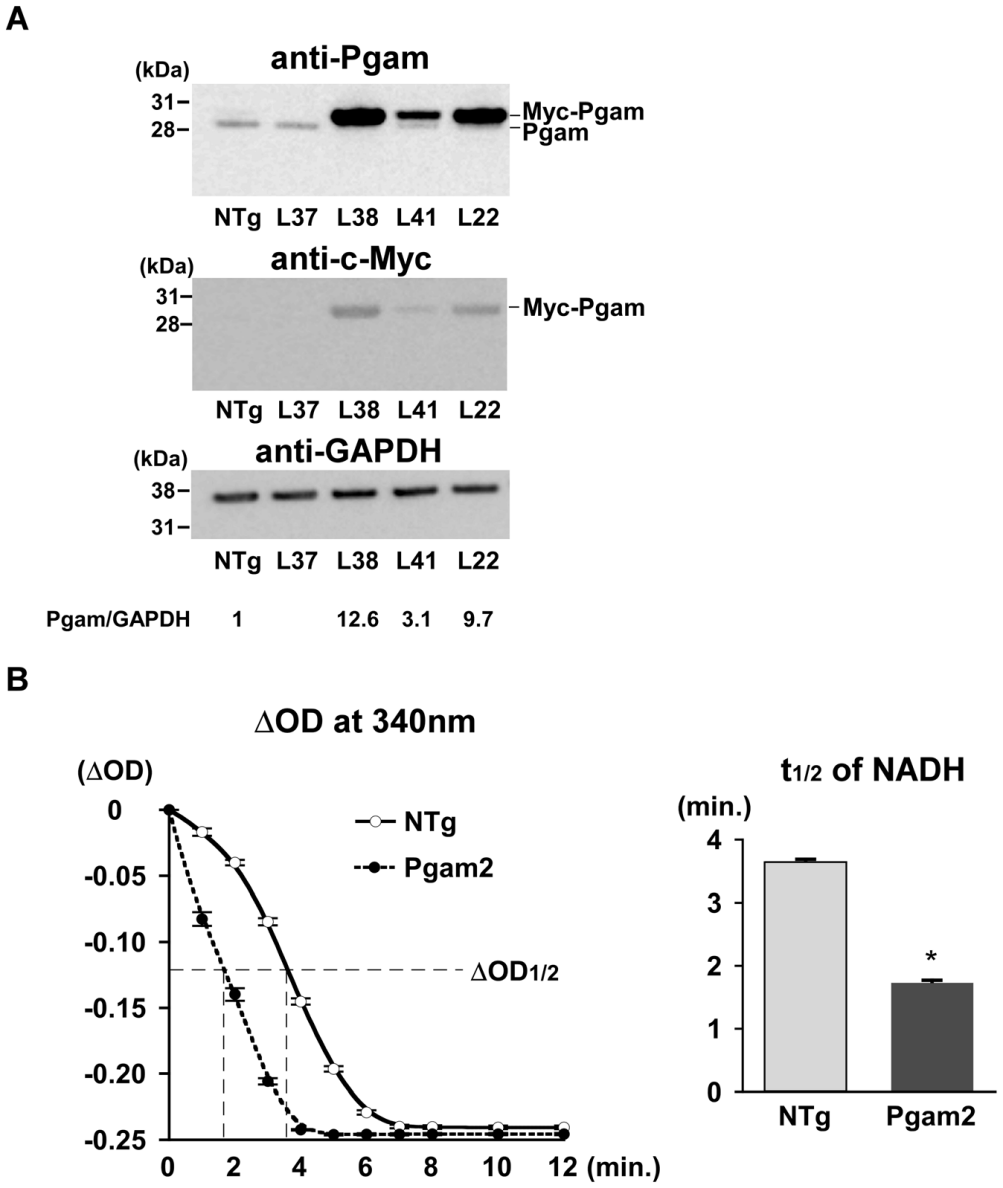
Generation of transgenic mice overexpressing Pgam2 in a heart-specific manner. (**A**) Transgenic mice overexpressing Pgam2 in a heart-specific manner were generated using the α-MHC promoter on a C57/BL6 background. The Pgam1 antibody (Abcam; Ab2220) detected both mouse PGAM1 and PGAM2 at similar sensitivities (Figure S1). Migration of the transgene product, the Pgam2 protein with a Myc-tag, was slower than that of the endogenous Pgam protein on a sodium dodecyl sulfate polyacrylamide gel electrophoresis (SDS-PAGE) gel. Two of the 3 lines of Pgam2 mice (line 22 (L22) and line 38 (L38)) expressed different total Pgam protein levels in the heart (9.7-fold and 12.6-fold, when measurements included endogenous Pgam relative to levels in non-transgenic (NTg) mice, respectively). (**B**) The enzymatic activity of phosphoglycerate mutase was analyzed via an NADH-consuming spectrophotometric assay. The vertical axis indicated differences in optical density (O.D.) at 340 nm from the value of the negative control without heart tissue lysate. The half-time of ΔO.D. at 340 nm was 53% lower in Pgam2 mice than in NTg mice (n = 4 for each group).

Transgenic mice overexpressing murine Pgam2 in a heart-specific manner were generated using the α-MHC promoter on a C57BL6 background. Five independently derived founders were produced from 43 screened mice. The progenies of 4 of the 5 founders expressed the transgene product as determined by PCR. The progeny of 3 of the 4 founders expressed the transgene product as determined by western blot analysis. The Pgam1 antibody detected both mouse PGAM1 and PGAM2 at a similar sensitivity (Figure S1). Migration of the transgene product, the Pgam2 protein with a Myc-tag, was slower than that of the endogenous Pgam protein on a sodium dodecyl sulfate polyacrylamide gel electrophoresis (SDS-PAGE) gel (Figure 1A). Two of the 3 lines of Pgam2 mice (line 22 (L22) and line 38 (L38)) expressed different total Pgam protein levels in the heart (9.7-fold and 12.6-fold, in which measurements included endogenous Pgam relative to levels in non-transgenic (NTg) mice, respectively). To confirm the accentuation of phosphoglycerate mutase enzymatic activity in Pgam2-transgenic mice, the half-time of absorbance at 340 nm to the completion of the reaction, which represented the amount of NADH consumed, was calculated in Pgam2 (L38) mice. The half-time of NADH was 53% shorter in Pgam2 (L38) mice than in NTg mice (*p<0.05 versus NTg; n = 4 each; Figure 1B). First, we analyzed L22 and L38. Both transgenic lines survived normally over a follow-up period of 1 year. Both lines showed normal cardiac function, determined by echocardiography at rest (Table 1), and normal heart weights (Table 2). Thereafter, Pgam2 (L38), which showed the highest expression levels of the Pgam protein, was subjected to further analysis, and was designated as Pgam2 mice unless otherwise specified.

**Table 1:** Echocardiographic analysis of Pgam2 mice.

	NTg			Pgam2 (L22)			Pgam2 (L38)		
	(n = 10)			(n = 10)			(n = 10)		
Heart rate (bpm)	545	±	13	533	±	14	541	±	9
LV diastolic diameter (mm)	3.62	±	0.05	3.89	±	0.04	3.44	±	0.04
LV systolic diameter (mm)	1.95	±	0.05	2.21	±	0.02	2.00	±	0.07
Fractional shortening (%)	44.7	±	0.7	43.2	±	2.9	41.9	±	1.9
Posterior wall thickness (mm)	0.67	±	0.06	0.53	±	0.01	0.67	±	0.07

Values are expressed as the mean ± SEM. NTg: non-transgenic mice; Pgam2: phosphoglycerate mutase 2 transgenic mice; bpm: beats per minute; LV: left ventricular.

**Table 2:** Pathological analysis of Pgam2 mice.

	NTg			Pgam2 (L22)			Pgam2 (L38)		
	(n = 10)			(n = 10)			(n = 10)		
Body weight (BW, g)	25.2	±	0.6	25.6	±	0.8	24.4	±	0.4
Heart weight (HW, mg)	123.6	±	3.4	121.3	±	2.9	115.4	±	2.5
HW/BW (mg/g)	5.03	±	0.06	4.89	±	0.06	4.79	±	0.07
Lung weight (LW, mg)	151.2	±	2.8	150.7	±	2.5	154.0	±	3.8
LW/BW (mg/g)	6.22	±	0.11	5.87	±	0.09	6.29	±	0.11

Values are expressed as the mean ± SEM. NTg: non-transgenic mice; Pgam2: phosphoglycerate mutase 2 transgenic mice.

### 2. Myocardial uptake of ^18^FDG or ^125^I-9MPA was normal in Pgam2 mice

**Figure 2:**
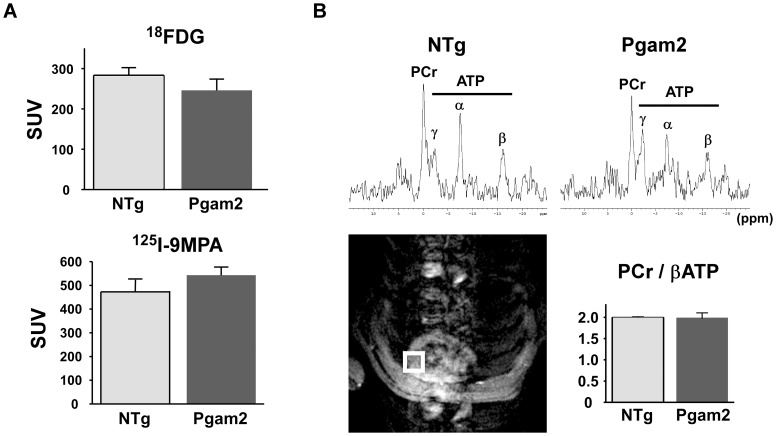
Myocardial substrate uptake and myocardial energy reserve were normal in Pgam2 mice. (**A**) Myocardial uptake of ^18^F-deoxyglucose (^18^FDG) and ^125^I-15-(*p*-iodophenyl)-9-*R,S*-methylpentadecanoic acid (^125^I-9MPA) did not differ from that in NTg mice (n = 13 for NTg and n = 18 for Pgam2 mice). SUV: standard uptake value. SUV = tissue concentration (MBq/g)/(injected dose (MBq)/body weight (g)). (**B**) Cardiac energy reserve was analyzed by measuring cardiac high-energy phosphates with *in situ* ^31^P magnetic resonance spectroscopy (MRS). ^1^H magnetic resonance (MR) imaging was used to define the region of interest to measure the ^31^P MR spectrum of the anterior wall of the left ventricle (lower left panel). Representative *in situ* cardiac ^31^P MR spectra from NTg and Pgam2 mice are shown (upper panel). ppm: parts per million. The cardiac phosphocreatine (PCr)/βATP ratio of Pgam2 mice at rest did not differ from that of NTg mice (NTg: n = 10; Pgam2 mice: n = 9; lower right panel).

To examine changes in substrate utilization in Pgam2 hearts, we examined the myocardial uptake of glucose and fatty acids using their analogs ^18^FDG and^ 125^I-9MPA, respectively. The uptake of ^18^FDG and ^125^I-9MPA in Pgam2 mice was similar to that in NTg control mice (Figure 2A, Table S2).

### 3. Myocardial energy reserve assessed by in situ ^31^P magnetic resonance spectroscopy (MRS) was normal in Pgam2 mice

The energy reserve of the heart was analyzed by measuring high-energy phosphates using *in situ* ^31^P magnetic resonance spectroscopy (MRS) [3]. The phosphocreatine (PCr)/βATP ratio, identified as a marker of myocardial energy reserve, was reported to be a prognostic indicator of heart failure [35]. The mean cardiac PCr/βATP ratio of NTg mice at rest was 2.0, which was consistent with a previous report [36]. The mean cardiac PCr/βATP ratio of Pgam2 mice at rest was not different from that of NTg mice (Figure 2B).

### 4. Metabolomic profile of Pgam2 mice

**Figure 3:**
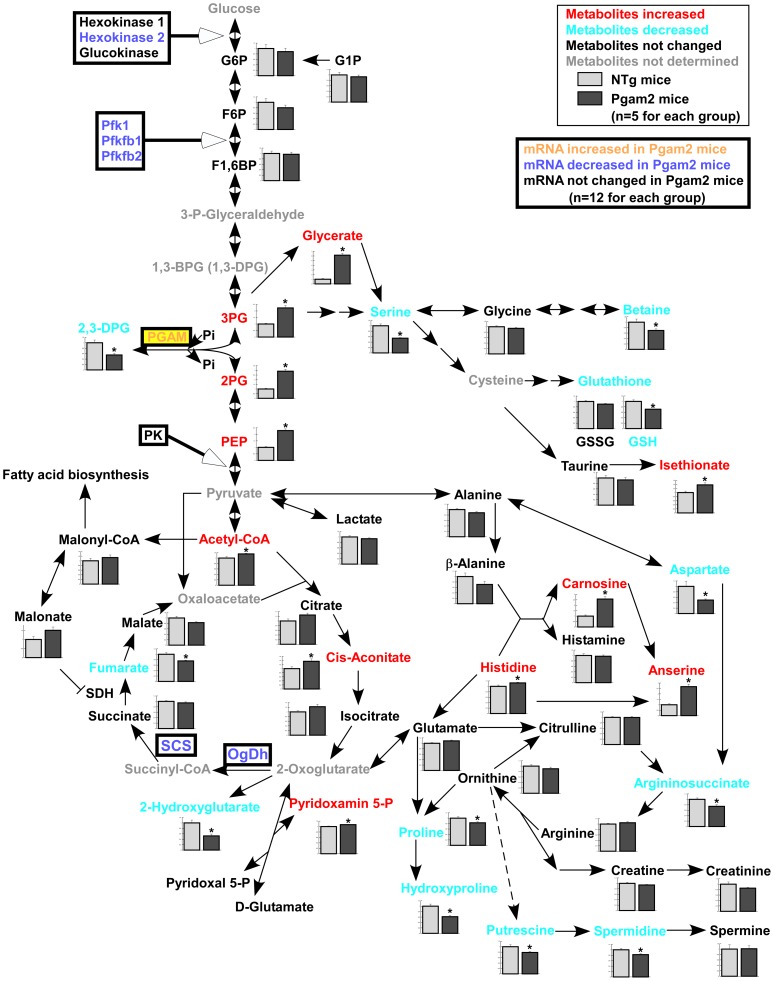
Metabolomic analysis revealed an alteration in the intermediates of glycolysis, the TCA cycle, and amino acids. Comprehensive metabolomic analysis revealed that levels of the upstream metabolites of phosphoglycerate mutase remained unchanged, while levels of the downstream metabolites, such as 2-phosphoglycerate and phosphoenolpyruvate, were increased. Lactate, the final metabolite of glycolysis, also remained unchanged. While metabolites of the first half of the TCA cycle, such as acetyl-CoA and cis-aconitate, were increased, those of the latter half, such as fumarate, were decreased (n = 5 for each group). The expressions of some genes involved in glycolysis and the TCA cycle, which are shown in Figure 4 or Figure 6, are also presented.

To examine the effect of the persistent overexpression of Pgam2 on myocardial energy metabolism, we performed a comprehensive metabolomic analysis. The results of the quantification of metabolites related to glycolysis and the TCA cycle, and amino acids and their derivatives, are listed in Table S3. Pgam2 overexpression significantly changed the metabolome of heart tissue (Figure 3 and Table S3). First, the levels of metabolites just upstream and downstream of Pgam were significantly changed, with 3-phosphoglycerate (3PG), 2-phosphoglycerate (2PG), and phosphoenolpyruvate (PEP) levels increasing, and 2,3-diphosphoglycerate (2,3-DPG) level decreasing. The levels of metabolites in the initial steps of the glycolytic pathway, such as glucose-6-phosphate (G6P), fructose-6-phosphate (F6P), and fructose-1,6-bisphosphate (F1,6BP), remained unchanged. Lactate, an end product of the glycolytic pathway, also did not change. Second, Pgam2 overexpression altered the levels of metabolites in the TCA cycle. The levels of metabolites in the first half of the TCA cycle were slightly higher, whereas acetyl-CoA and cis-aconitate were significantly higher. The levels of metabolites in the latter half of the cycle were slightly lower, whereas fumarate was significantly lower. Third, the levels of several amino acids and their derivatives were changed. For example, serine, betaine, the reduced-form glutathione (GSH), and aspartate decreased, while histidine, carnosine, and anserine increased.

### 5. Overexpression of Pgam2 suppressed the expression of genes related to glycolysis, and inhibited phosphofructokinase activity

**Figure 4:**
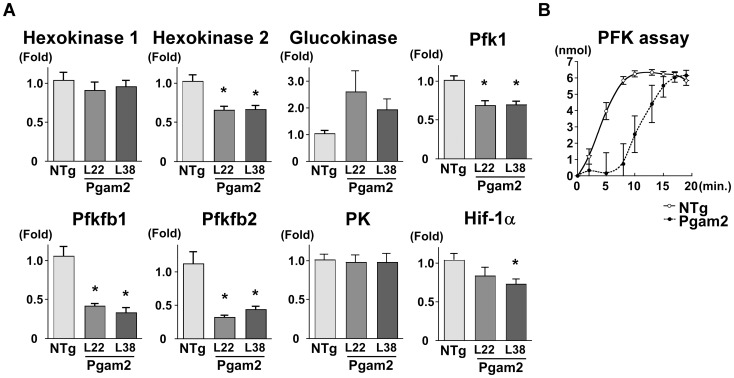
The expression of genes related to glycolysis and phosphofructokinase (PFK) enzymatic activity were modified. (**A**) The expression of genes related to glycolysis was analyzed using quantitative real-time PCR. Hexokinase 2, 6-phosphofructo-2-kinase/fructose-2,6-biphosphatase 1 (Pfkfb1), 6-phosphofructo-2-kinase/fructose-2,6-biphosphatase 2 (Pfkfb2), and phosphofructokinase 1 (Pfk1) levels were decreased in Pgam2 mice. The amount of target gene mRNA was normalized by 18S rRNA mRNA. Values are the mean ± SEM. Gene expression levels in Pgam2 mice were compared with those of NTg mice. *p<0.05 versus NTg mice (n = 12 for each group). (**B**) Measuring PFK enzymatic activity. Pgam2 overexpression inhibited myocardial PFK activity. The vertical axis indicated the amount of NADH produced, which represented PFK activity (NTg: n = 6, Pgam2 mice: n = 5).

The overexpression of Pgam2 did not change the uptake of the glucose analogs, G6P and lactate, which indicated that the glycolytic flux may not be increased. Thus, we hypothesized that the persistent overexpression of Pgam2 may modify other enzymes in the glycolytic pathway. First, we examined the effect of Pgam2 overexpression on the gene expression of other rate-limiting glycolytic enzymes by quantitative real-time PCR. The gene expression levels of hexokinase 2, phosphofructokinase 1 (Pfk1), 6-phosphofructo-2-kinase/fructose-2,6-biphosphatase 1 (Pfkfb1), and 6-phosphofructo-2-kinase/fructose-2,6-biphosphatase 2 (Pfkfb2), the rate-limiting enzymes in glycolysis, were decreased (Figure 4A). The gene expression levels of hypoxia-inducible factor 1 α subunit (Hif-1α), a key transcription factor and positively regulates glycolytic enzymes [37], were decreased in Pgam2 mice. Since the increase in 3-phosphoglycerate, 2-phosphoglycerate, or phosphoenolpyruvate each resulted in the inhibition of phosphofructokinase [38], we measured the enzymatic activity of phosphofructokinase (PFK) using heart tissue lysates. The activity of PFK was significantly decreased in Pgam2 mice (Figure 4B). Thus, Pgam2 overexpression decreased the expression of genes related to glycolysis and PFK activity.

### 6. Overexpression of Pgam2 decreased the capacity for respiration and increased that for the generation of reactive oxygen species in vitro

**Figure 5:**
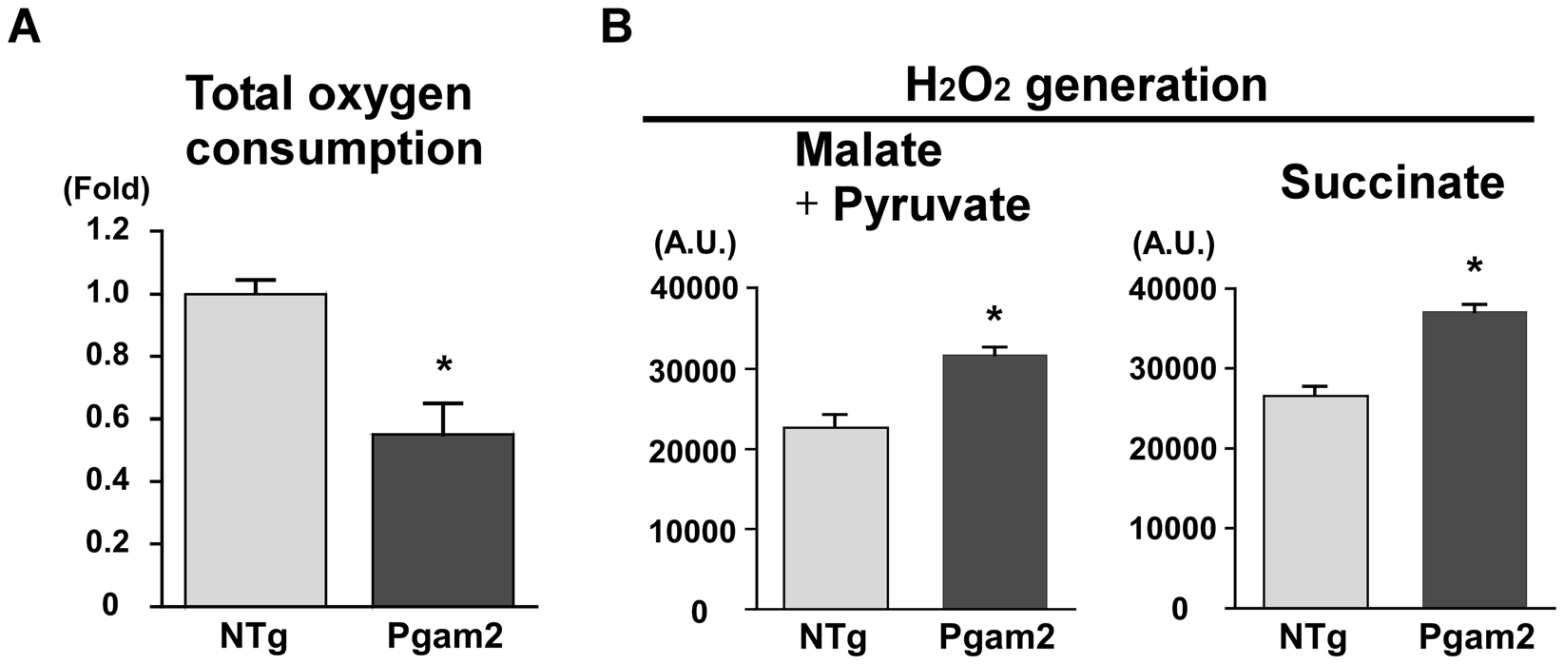
Respiration was decreased and ROS production was increased in the isolated mitochondria of Pgam2 mice. (**A**) Oxygen consumption in isolated mitochondria was measured using an oxygen electrode cuvette. Total oxygen consumption was lower in Pgam2 mice. Values are the mean ± SEM. *p<0.05 versus NTg mice (NTg: n = 3; Pgam2 mice: n = 5). (**B**) Generation of H_2_O_2_ by isolated mitochondria. Malate and pyruvate, or succinate, were used as substrates. The generation of H_2_O_2_ increased in Pgam2 mice both with malate + pyruvate and with succinate. Values are the mean ± SEM. *p<0.05 versus NTg mice (n = 6 for each group). A.U.: arbitrary unit.

Since the levels of metabolites in the TCA cycle were changed and the function of the TCA cycle is closely linked to that of mitochondria, we examined the capacity for respiration and generation of reactive oxygen species (ROS) in mitochondria isolated from heart tissue. Oxygen consumption by isolated mitochondria measured *in vitro* was lower in Pgam2 mice (Figure 5A). The amount of H_2_O_2_ generated from isolated mitochondria measured *in vitro* was increased (Figure 5B). Thus, the regulation of respiration and ROS generation in mitochondria were impaired in Pgam2 mice.

### 7. Overexpression of Pgam2 suppressed the expression of genes related to mitochondria

**Figure 6:**
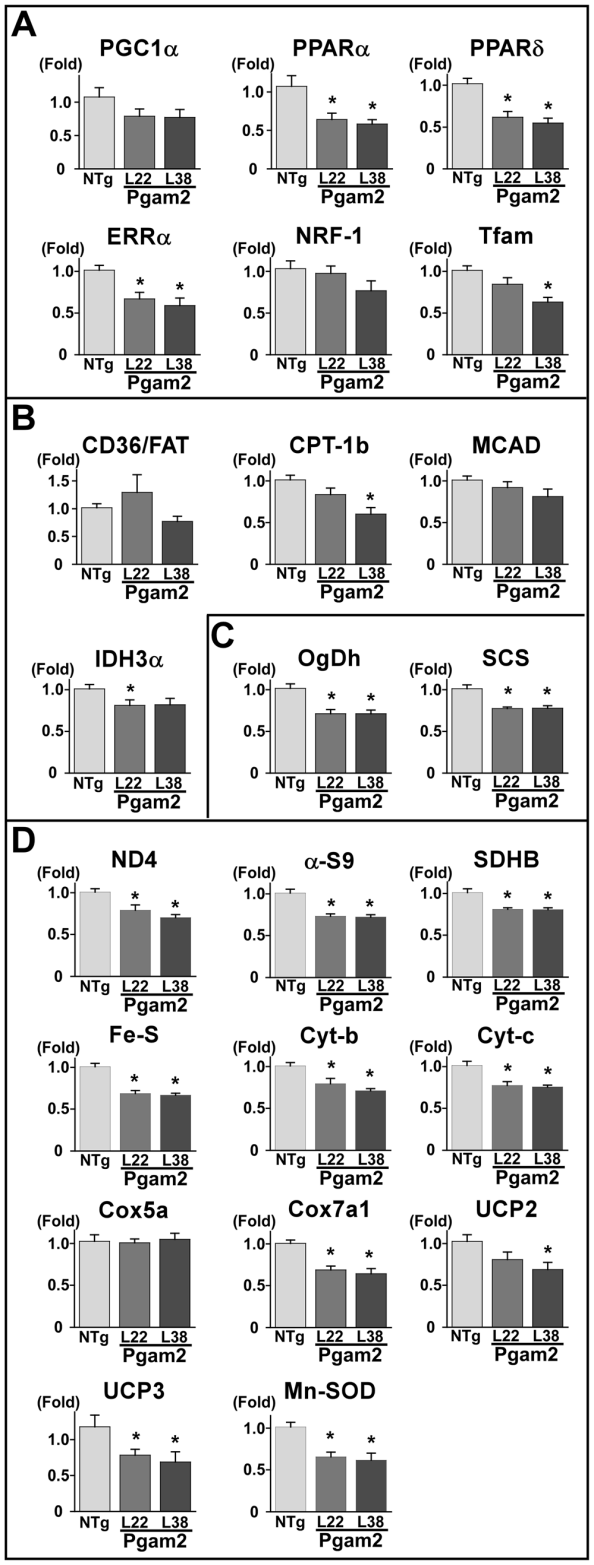
The expression of genes related to mitochondria was decreased in Pgam2 mice. The expression of genes related to mitochondria was analyzed using quantitative real-time PCR. The genes presented here are involved in **(A)** transcriptional regulators, **(B)** fatty acid metabolism, **(C)** the TCA cycle, and **(D)** mitochondria. Peroxisome proliferator-activated receptor α (PPARα), peroxisome proliferator-activated receptor δ (PPARδ), estrogen related receptor α (ERRα), mitochondrial transcription factor A (Tfam), carnitine palmitoyltransferase 1b (CPT-1b), isocitrate dehydrogenase 3 α (IDH3α), oxoglutarate dehydrogenase (OgDh), succinyl-CoA synthetase α (SCS), mitochondrially encoded NADH dehydrogenase 4 (ND4), alpha-subcomplex 9 of complex I (α-s9), mitochondrial succinate dehydrogenase iron-sulfur subunit (SDHB), iron-sulfur protein (Fe-S), cytochrome b (Cyt-b), cytochrome c (Cyt-c), cytochrome c oxidase subunit VIIa (COX7a), uncoupling protein 2 (UCP2), uncoupling protein 3 (UCP3), and manganese superoxide dismutase (Mn-SOD) levels were decreased in Pgam2 mice. NRF-1: nuclear respiratory factor 1; CD36/FAT: CD36/fatty acid translocase; MCAD: medium-chain acyl coenzyme A dehydrogenase; Cox5a: mitochondrial cytochrome c oxidase subunit Va. The amount of target gene mRNA was normalized by 18S rRNA mRNA. Values are the mean ± SEM. Gene expression levels in Pgam2 mice were compared with those of NTg mice. *p<0.05 versus NTg mice (n = 12 for each group).

Since respiration and ROS generation were altered in isolated mitochondria, we quantified the expression of genes related to mitochondrial function. Peroxisome proliferator-activated receptor γ coactivator 1-α (PGC-1α) is a master regulator of mitochondrial function and biogenesis, and regulates the expression of peroxisome proliferator-activated receptor α (PPARα) and PPARδ, estrogen-related receptor α (ERRα), nuclear respiratory factor-1 (NRF-1), and mitochondrial transcription factor A (Tfam). The gene expression levels of PPARα decreased, accompanied by a decrease in enzymes involved in fatty acid metabolism, such as carnitine palmitoyltransferase-1b (CPT-1b) and isocitrate dehydrogenase (IDH3α) (Figure 6A, 6B). The gene expression levels of ERRα, PPARδ, and Tfam also decreased (Figure 6A). The gene expression levels of enzymes in the TCA cycle, such as oxoglutarate dehydrogenase (OgDh) and succinyl-CoA synthetase (SCS), were decreased (Figure 6C), and this decrease may explain the accumulation of TCA cycle metabolites, such as acetyl-CoA and cis-aconitate. The expression levels of genes in the mitochondrial respiratory chain, such as mitochondrially encoded NADH dehydrogenase 4 (ND4), alpha-subcomplex 9 of complex I (α-S9), succinate dehydrogenase b (SDHB), iron-sulfur protein (Fe-S), cytochrome b (Cyt-b), cytochrome c (Cyt-c), and mitochondrial cytochrome c oxidase subunit VIIa 1 (Cox7a1), were decreased. The gene expression levels of uncoupling protein (UCP) 2, UCP3, and manganese superoxide dismutase (MnSOD) were also decreased (Figure 6D).

### 8. Mitochondrial morphology and a marker of ROS in vivo were normal in Pgam2 mice

A light microscopic examination revealed that no cardiomyopathic changes, such as necrosis or fibrosis, were observed in Pgam2 mice (data not shown). Electron-microscopic analysis showed no apparent abnormalities. The size and number of mitochondria did not differ between Pgam2 (H) mice and their NTg littermates. Neither the degeneration nor collapse of mitochondria was observed (Figure S2A). We then measured TBARS levels, a marker of lipid peroxidation, in heart tissue. TBARS levels were normal in the heart tissue of Pgam2 mice (Figure S2B), which indicated that ROS generation was not increased *in vivo* at the baseline.

### 9. Cardiac function in response to dobutamine was impaired in Pgam2 mice

**Figure 7:**
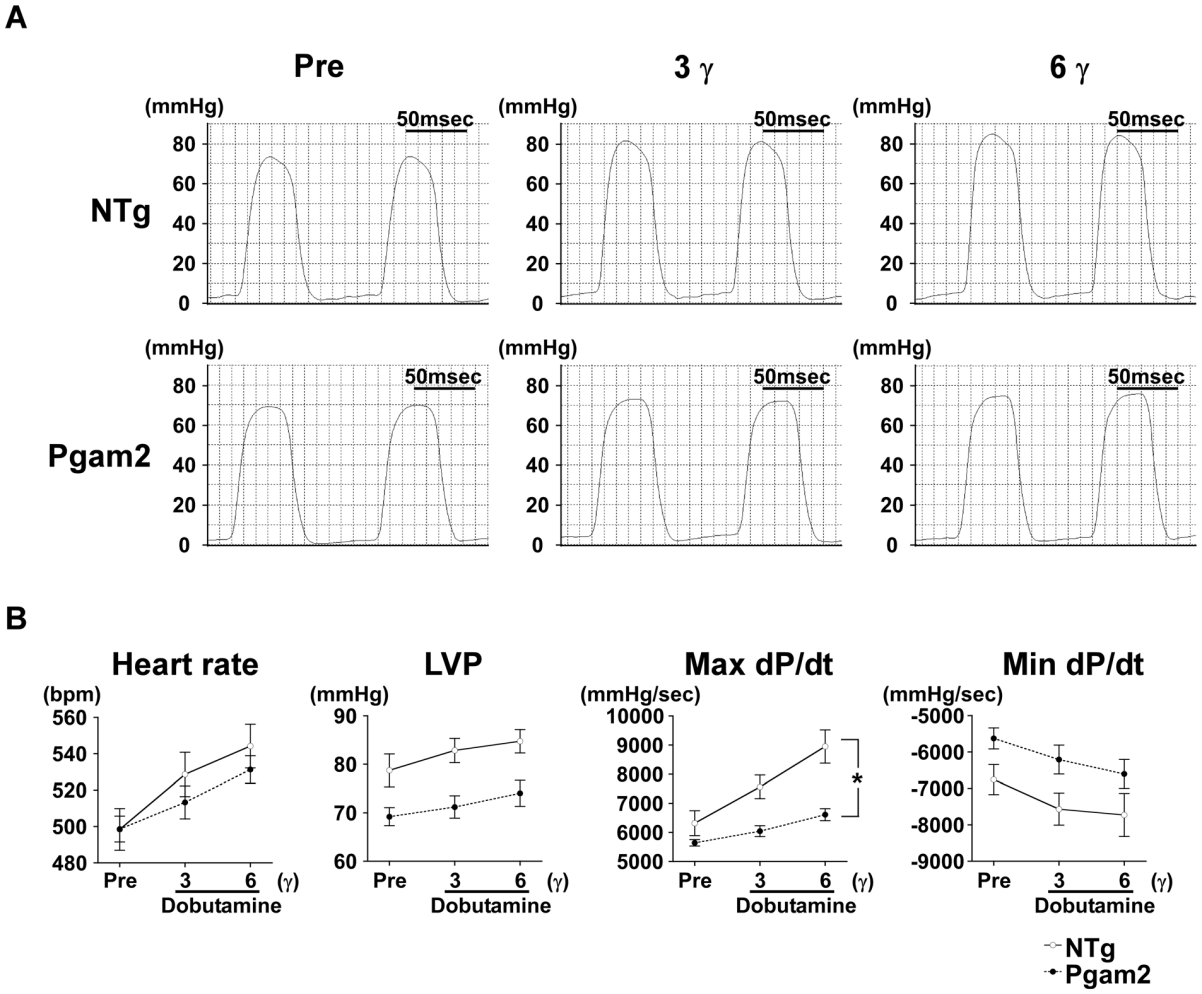
Cardiac function in response to dobutamine was impaired in Pgam2 mice. Cardiac function was analyzed by cardiac catheterization under dobutamine infusion. (**A**) Representative traces of left ventricular pressures. (**B**) Cardiac systolic function in response to dobutamine was impaired in Pgam2 mice. LVP: left ventricular systolic pressure; γ: μg/kg/min. A two-way repeated-measures ANOVA was used to test differences between groups in response to dobutamine infusion. Values are the mean ± SEM. *p<0.05, interaction of dobutamine doses with differences between Pgam2 mice and NTg mice (n = 10 for each group).

As described, cardiac function at rest as assessed by echocardiography was normal in Pgam2 mice. However, the expression levels of genes related to mitochondrial function were decreased and the capacity of mitochondrial function assessed *in vitro* was perturbed in Pgam2 mice. Since the decrease in gene expression levels related to mitochondrial function was previously shown to be associated with the decrease in contractile reserve under dobutamine infusion [39], we assessed cardiac function using cardiac catheterization under graded doses of dobutamine infusion. The change in maximum dP/dt, a marker of cardiac systolic function, in response to dobutamine was blunted in Pgam2 mice (Figure 7A, 7B).

### 10. Pgam2 mice developed systolic dysfunction and myocardial fibrosis in response to pressure overload

**Figure 8:**
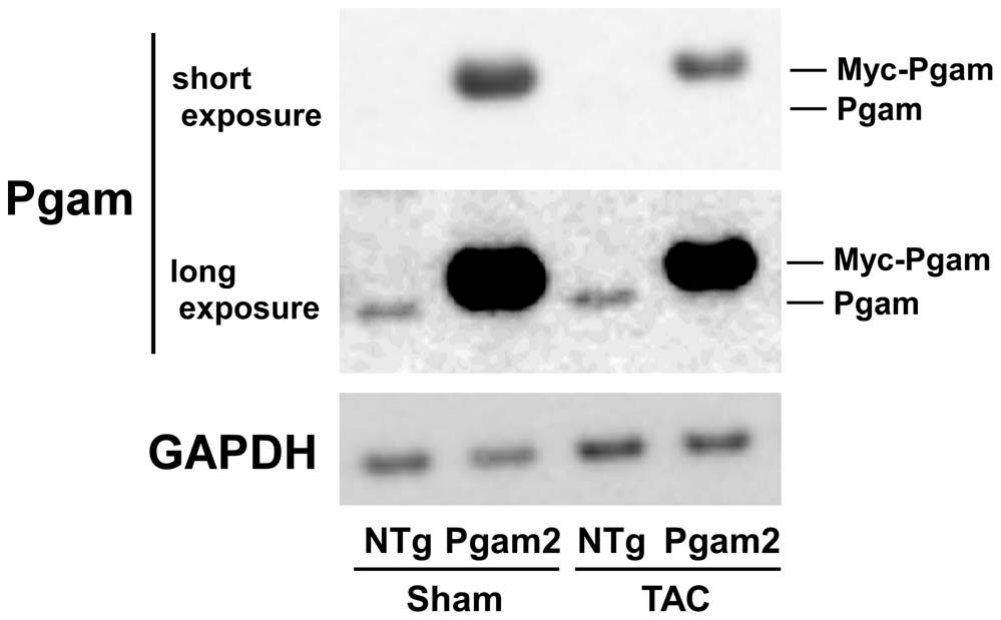
Pgam protein in NTg or Pgam2 mice with the sham or TAC operation. A representative western blot of NTg or Pgam2 mice with the sham or transverse aortic constriction (TAC) operation.

To further examine the cardiac function of Pgam2 mice under stress conditions, we examined the effect of Pgam2 overexpression on pressure overload. NTg or Pgam2 mice were subjected to transverse aortic constriction (TAC) or a sham operation at 3 months of age, and were analyzed 14 days after the operation. Pgam protein levels in NTg mice with TAC were not different from those in NTg mice with the sham operation (Figure 8 and Table 3). Pgam protein levels in Pgam2 mice with TAC were 6.9-fold higher than those in NTg mice with TAC.

**Table 3:** Expression of the Pgam protein in NTg or Pgam2 mice with the sham operation or transverse aortic constriction.

	Sham		TAC	
	NTg	Pgam2	NTg	Pgam2
	(n = 4)	(n = 4)	(n = 4)	(n = 4)
Pgam/GAPDH	1.0 ± 0.4	13.1 ± 2.3^†^	0.7 ± 0.1	4.6 ± 0.6^*†^

Values are expressed as the mean ± SEM. NTg: non-transgenic mice; Pgam2: phosphoglycerate mutase 2 transgenic mice; TAC: transverse aortic constriction. The mean value of NTg mice with the sham operation was used as a standard. *p<0.05 versus the same genotype with the sham operation. †p<0.05 versus NTg mice with the same operation.

**Figure 9:**
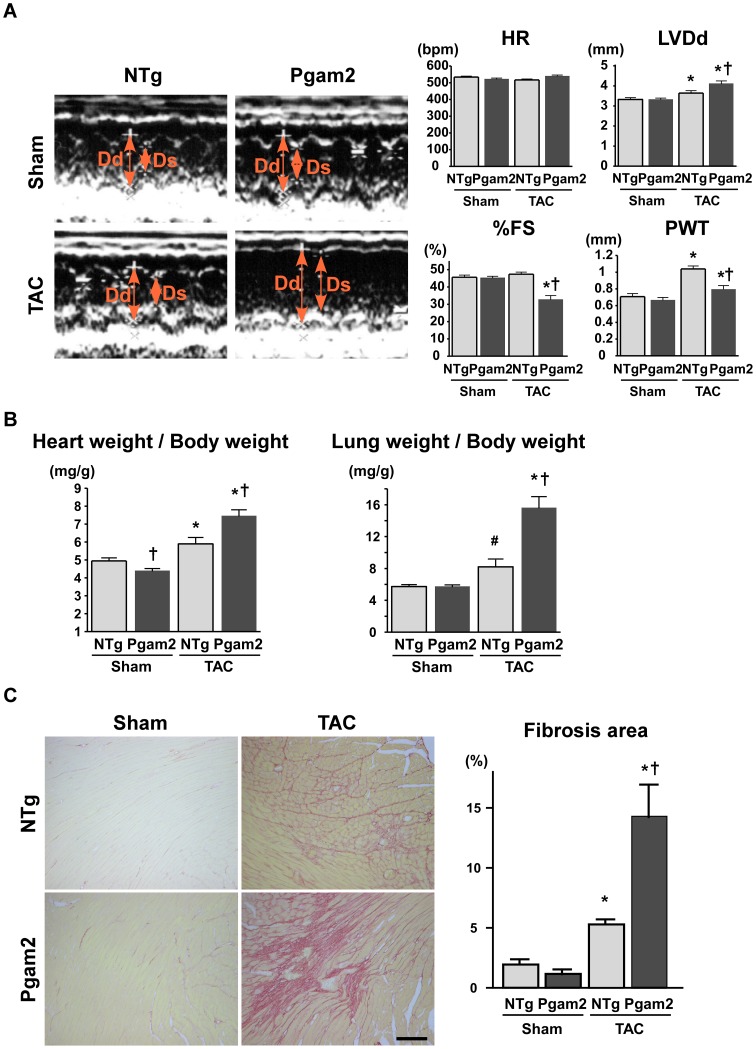
Pgam2 mice developed systolic dysfunction and myocardial fibrosis in response to pressure overload. (A) A representative M-mode echocardiogram is shown in the left panel. The left ventricular end-diastolic dimension (LVDd) was higher and %FS was lower in Pgam2 mice with TAC than in NTg mice with TAC. Dd: LVDd; Ds: left ventricular end-systolic dimension; HR: heart rate; bpm: beats per minute; FS: fractional shortening; PWT: posterior wall thickness. (B) The heart weight/body weight ratio and lung weight/body weight ratio of Pgam2 mice were higher than those of NTg mice with TAC. (C) Myocardial fibrosis was analyzed using Sirius Red staining. The ratio of the fibrotic area to the whole short-axis sectional area was calculated. Myocardial interstitial fibrosis was observed in NTg mice with TAC. Myocardial fibrosis was enhanced in Pgam2 mice with TAC. Values are the mean ± SEM. *p<0.05 versus the same genotype with the sham operation. †p<0.05 versus NTg mice with the same operation. # p = 0.08 vs NTg mice with the sham operation. Sham-operated NTg mice: n = 6; sham-operated Pgam2 mice: n = 4; NTg mice with TAC: n = 8; Pgam2 mice with TAC: n = 6.

The systolic function of the heart, as assessed by fractional shortening (FS), was normal in NTg mice and Pgam2 mice with the sham operation. Upon TAC, NTg mice developed cardiac hypertrophy with preserved systolic function. However, Pgam2 mice with TAC developed systolic dysfunction (Figure 9A). The heart weight/body weight ratio (HW/BW) of Pgam2 mice was higher than that of NTg mice with TAC (Figure 9B). The lung weight (LW)/body weight ratio (LW/BW) of Pgam2 mice was also higher than that of NTg mice with TAC (Figure 9B), which suggested the presence of pulmonary congestion in Pgam2 mice with TAC. Fibrosis was not observed with Sirius Red staining in Pgam2 mice with the sham operation. However, the fibrotic area in Pgam2 mice with TAC was significantly larger than that of NTg mice with TAC (Figure 9C). Thus, Pgam2 mice developed heart failure associated with enhanced cardiac hypertrophy and fibrosis.

**Figure 10:**
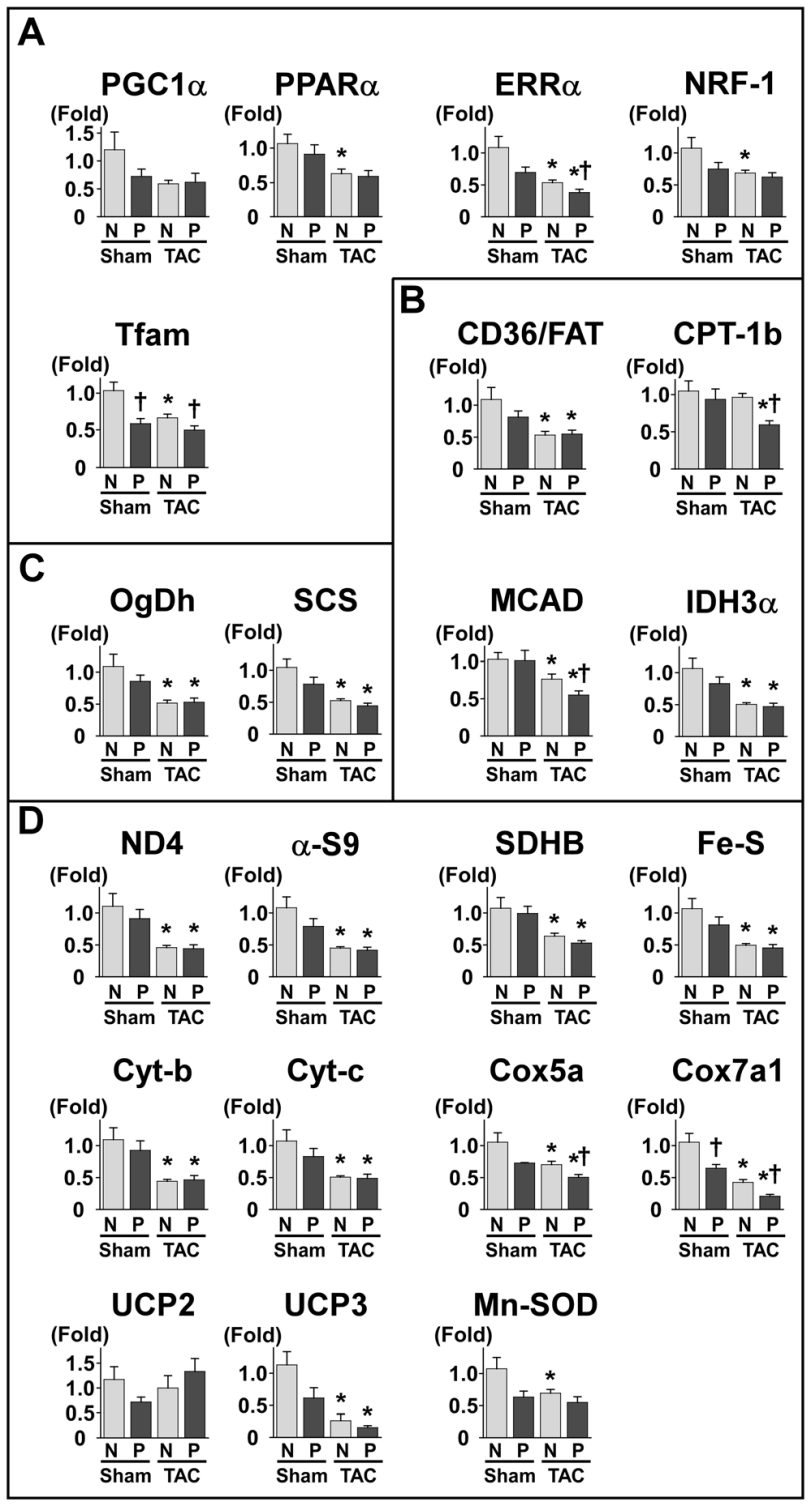
The expression of genes related to mitochondrial function under the TAC operation. The expression of genes related to mitochondrial energy metabolism was decreased in NTg mice with TAC. The genes presented here are (**A**) transcriptional regulators, (**B**) fatty acid metabolism, (**C**) the TCA cycle, and (**D**) mitochondria. Pgam2 overexpression further decreased the expression of some of these genes, such as ERRα, Tfam, MCAD, Cox5a, and Cox7a1 with TAC. Values are the mean ± SEM. N: NTg mice. P: Pgam2 mice. *p<0.05 versus the same genotype with the sham operation. †p<0.05 versus NTg mice with the same operation. Sham-operated NTg mice: n = 8; sham-operated Pgam2 mice: n = 8; NTg mice with TAC: n = 10; Pgam2 mice with TAC: n = 14.

We then examined the expression of genes related to mitochondria in NTg and Pgam2 mice under sham and TAC operations. The expression of several genes related to mitochondria was decreased in NTg mice with TAC (Figure 10A-D). Pgam2 overexpression further decreased the expression of some genes, such as ERRα, Tfam, medium-chain acyl coenzyme A dehydrogenase (MCAD), Cox5a, and Cox7a1, in response to TAC (Figure 10A-D).

## Discussion

We examined the effects of the persistent overexpression of Pgam2 on energy metabolism and stress resistance in the heart in this study. Cardiac function at rest was normal. Uptake of the analogs of glucose and a fatty acid, and the PCr/βATP ratio at rest were normal in Pgam2 mice. However, the persistent overexpression of Pgam2 altered the levels of metabolites involved in glycolysis and the TCA cycle, and the expression of genes related to mitochondrial function at baseline. The capacity for mitochondrial respiration decreased, and that for mitochondrial ROS production increased in *in vitro* experiments using isolated mitochondria. Pgam2 mice developed systolic dysfunction upon dobutamine infusion and pressure overload.

The Pgam protein in Pgam2 mice with TAC was 6.9-fold higher than that in NTg mice with TAC. Pgam protein levels in Pgam2 mice with TAC were lower than those in Pgam2 mice with the sham operation, which may have been due to reduced α-MHC promoter activity upon TAC [40]. Pgam2 mice with the sham operation showed preserved cardiac function. Cardiac function in NTg mice with TAC was preserved. However, Pgam2 mice with TAC showed decreased systolic function and increased lung weight, which indicated the development of heart failure. Pgam protein levels were shown to increase by approximately 5-fold in a canine model of heart failure [24]. Thus, increased Pgam protein levels were involved in the development of heart failure under stressed conditions.

Pgam2 overexpression in primary mouse embryonic fibroblasts (MEFs) increased the production of ^3^H_2_O from [3-^3^H]glucose and lactate production, which indicates that glycolytic flux is increased [18]. Although we did not measure the actual glycolytic flux, we suggest that it was not changed in Pgam2 hearts for the following reasons. First, uptake of the glucose analog and G6P was not changed, which indicates that the level of glucose that enters the glycolytic pathway may not be changed. Second, although the levels of metabolites just upstream and downstream of Pgam were changed, those in the initial steps of glycolysis and of lactate, an end product of glycolysis, remained unchanged. The intermediary metabolites of glycolysis, such as 3PG, 2PG, and PEP, were shown to be increased, and 3PG, 2PG, and PEP have been reported to inhibit PFK activity in plants [38]. Indeed, PFK activity was decreased and may have canceled the increase in Pgam enzymatic activity. The decrease in the gene expression of the rate-limiting enzymes of glycolysis may have also attenuated the booster effects of Pgam2 overexpression on glycolytic flux. The different effects of Pgam2 overexpression on glucose metabolism in MEFs and murine hearts may be due to metabolic differences between cultured fibroblasts and intact heart tissue.

The major biological function of mitochondria is ATP synthesis via oxidative phosphorylation. In addition, mitochondria play an important role in various cellular functions including redox homeostasis, calcium regulation, apoptosis, and the synthesis and catabolism of metabolites. A number of *in vitro* and *in vivo* methods have been used to examine the various functions of mitochondria, and have advantages and limitations [41], [42]. The capacity for ROS generation measured *in vitro* using isolated mitochondria was increased, while TBARS, a marker of ROS measured *in vivo*, did not change in Pgam2 mice. Mitochondrial morphology was also normal on electron microscopic analysis. Differences in the markers of ROS between *in vitro* and *in vivo* analyses may be explained by other cellular factors that may have regulated the mitochondrial functions observed *in vivo*, over those observed *in vitro*. In addition, the abnormal functions of isolated mitochondria in the presence of normal mitochondrial morphology have been previously reported [43]. We speculate that, in spite of relatively normal morphology, persistent Pgam2 overexpression perturbed some of the functions of mitochondria and increased susceptibility to stress.

The mechanism by which Pgam2 overexpression impairs mitochondria is unknown and requires further investigation. However, Pgam2 overexpression also reduces mitochondrial respiration in MEFs [44]. One possibility is that the accumulation of glycolytic and TCA cycle intermediates caused mitochondrial dysfunction. Phosphoenolpyruvate (PEP) was shown to inhibit mitochondrial respiration [45], and was 2.3-fold higher in Pgam2 mice. Mitochondrial complex II is composed of ubiquinone oxidoreductase and succinate dehydrogenase (SDH), which oxidizes succinate to fumarate, reduces ubiquinone, and connects the TCA cycle and mitochondrial respiration. TCA cycle intermediates are known to regulate complex II activity and ROS generation [46]. Amino acids have also been shown to regulate mitochondrial function. The depletion of endogenous GSH increased mitochondrial ROS [47] and decreased mitochondrial respiration [48]. Aspartate and betaine protected cardiac mitochondrial function in a rat model of myocardial infarction [49], [50]. Proline and betaine were also shown to protect mitochondrial electron transport chain Complex II in plants [51]. GSH, aspartate, betaine, proline, and spermidine levels were decreased in Pgam2 mice.

Furthermore, the mechanism by which Pgam2 overexpression changed the gene expression of mitochondrial proteins is currently unknown. However, communication between mitochondria and the nucleus may influence many cellular activities [52], and has been referred to as “mitochondrial retrograde signaling”. Signaling pathways known to be involved in this communication are the target of rapamycin (TOR) and calcium signaling. Thus, we speculate that mitochondrial retrograde signaling may be involved in the impaired gene expression of mitochondrial protein; however, further studies are required.

The effects of the persistent overexpression of Pgam2 on fatty acid metabolism are less clear because our metabolomic analysis was unable to measure lipids and fatty acids. However, it is likely that the persistent overexpression of Pgam2 modified fatty acid metabolism. The gene expression of CD36/fatty acid translocase (CD36/FAT) was normal in Pgam2 mice (Figure 6B), which was consistent with the normal uptake of ^125^I-9MPA (Figure 2A and Table S2). However, the gene expression of CPT-1b, which is necessary for the uptake of fatty acids by mitochondria, was decreased. In addition, gene expression levels of PPARα, PPARδ, and ERRα were decreased in Pgam2 mice (Figure 6A).

The heart oxidizes the most efficient fuel for respiration in order to adapt to changes in cardiac workload, oxygen supply, and substrate availability in an appropriate manner. The normal heart exhibits such “metabolic flexibility”, the loss of which is hypothesized to be involved in the development of heart failure [53]. In this study, the persistent overexpression of Pgam2 may have modified glucose, fatty acid, and mitochondrial metabolism, impaired metabolic flexibility and predispose Pgam2 mice to cardiac dysfunction under stressed conditions, such as dobutamine infusion and pressure overload.

Myocardial fibrosis was shown to be induced by several conditions, such as mechanical stress, myocardial ischemia, and inflammation [54]. It has recently become clear that energy metabolism in the cell is closely linked to inflammation [55]. For example, proinflammatory macrophages show a shift from oxidative phosphorylation to glycolysis, which mimics the metabolic change known as the Warburg effect in cancer cells. Mitochondrial dysfunction has been shown to induce inflammatory responses [56], [57]. In addition, perturbations in energy metabolism predispose the heart to significant myocardial fibrosis under stressed conditions [58], [59]. Thus, we suggest that a modification in energy metabolism by persistent Pgam2 overexpression may be one of the causes of the significant fibrosis observed in response to pressure overload.

### Limitations of this study

We did not directly measure glycolytic flux, and, instead, estimated the flux from the results of metabolomic analysis and uptake of a glucose analog. In addition, the findings of the present study need to be interpreted with caution when we consider the role of Pgam in cardiac physiology and pathophysiology for the following reasons. First, a large amount of the Pgam protein was persistently overexpressed, which caused secondary changes that may not be related to the function of Pgam2 itself. Second, the parameters of cardiac energy metabolism, such as the phosphocreatine/βATP ratio, substrate uptake, metabolite quantification, and mitochondrial functions were not analyzed under stressed conditions. Thus, the physiological and pathophysiological roles of Pgam should be investigated in more detail in the future using the inducible overexpression of Pgam2, Pgam2 knockout mice, or inhibitors of Pgam.

## Supporting Information

Figure S1 **The antibody against Pgam1 also served as that against Pgam2 with a similar sensitivity.** FLAG-tagged murine Pgam1 or Pgam2 was transfected into murine embryonic fibroblasts defective in p53. Total cellular lysates were analyzed by western blotting. The antibodies used were anti-Pgam1 (1∶1000; Abcam, Cambridge, UK; Ab2220) and anti-FLAG (1∶1000; Sigma, St. Louis, MO, USA; F1804). The similar sensitivities of the anti-Pgam1 antibody against the two isoforms of Pgam proteins were confirmed.
(TIF)

Figure S2 **Mitochondrial morphology and a marker of ROS were normal in the heart tissue of Pgam2 mice. (A)** Ultrastructural analysis of Pgam2 mice. Electron micrographs of histological sections of the left ventricle prepared from 12-week-old Pgam2 mice and non-transgenic littermates. The insets on the right panel are high-magnification images of the indicated portions (squares) of the images on the left. The morphology of mitochondria in Pgam2 mice was normal. Nucl.: nucleus. The bar represents 1 µm. The density (number per 100 µm^2^) and the size (μm^2^) of mitochondria within a cardiomyocyte were observed by electron microscopy (lower panels). Values are the mean ± SEM (n = 3 for each groups). **(B)** TBARS as a marker of oxidative stress. Thiobarbituric acid reactive substances (TBARS) levels were normal in the heart tissue of Pgam2 mice. Values are the mean ± SEM. NTg mice: n = 7; Pgam2 mice: n = 8.
(TIF)

Table S1 Primer sequences used for real-time quantitative RT-PCR.
(DOC)

Table S2 Myocardial uptake of ^18^FDG and ^125^I-9MPA.
(DOC)

Table S3 Concentrations of metabolites in the heart identified by metabolomic analysis.
(DOC)
