# Supplementary figures and images for: Persistent Overexpression of Phosphoglycerate Mutase, a Glycolytic Enzyme, Modifies Energy Metabolism and Reduces Stress Resistance of Heart in Mice
Source: PLoS One. 2013 Aug 12;8(8):e72173. doi: 10.1371/journal.pone.0072173 (PMC3741204; doi:10.1371/journal.pone.0072173)

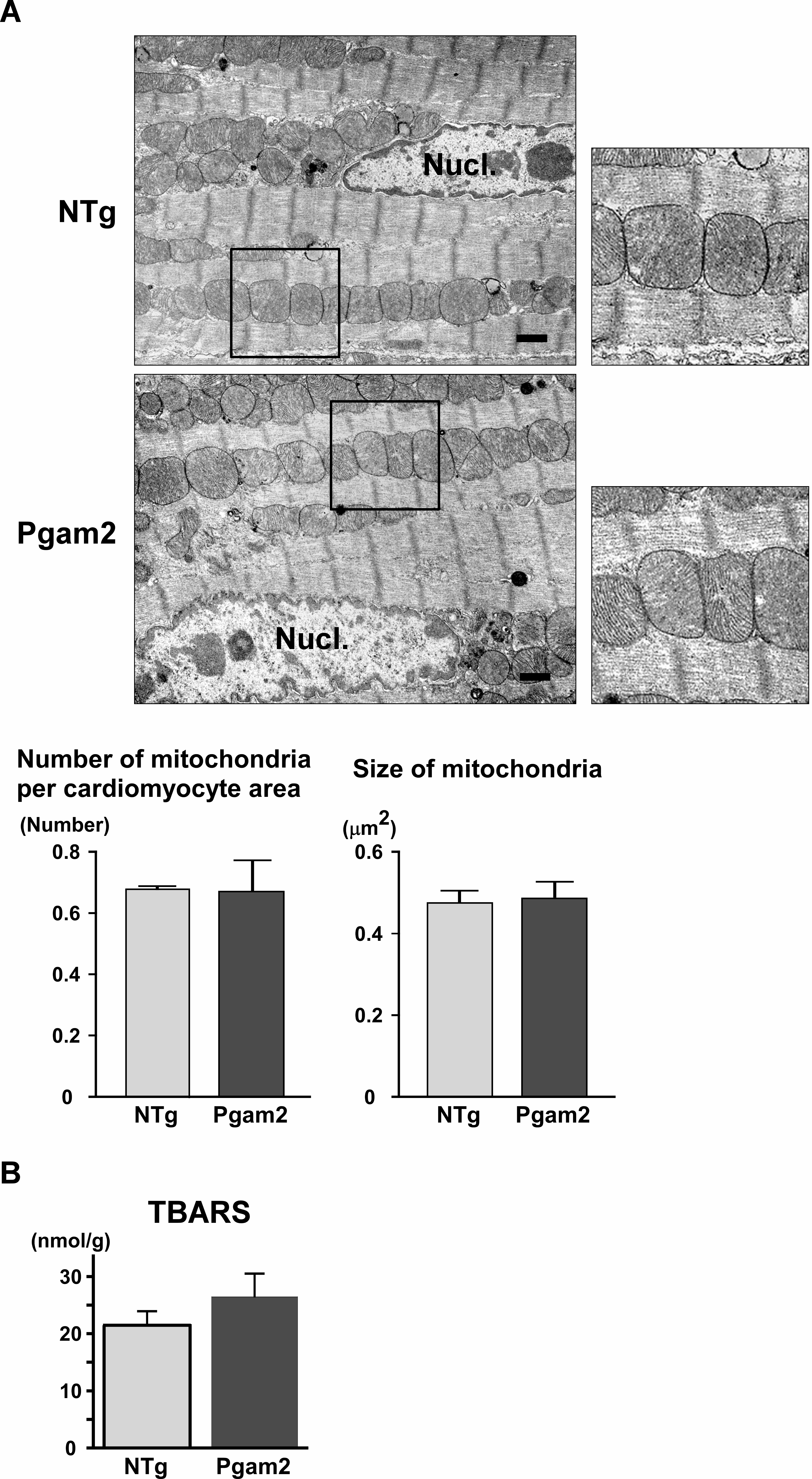

Supplement: Figure S2 — Mitochondrial morphology and a marker of ROS were normal in the heart tissue of Pgam2 mice. (A) Ultrastructural analysis of Pgam2 mice. Electron micrographs of histological sections of the left ventricle prepared from 12-week-old Pgam2 mice and non-transgenic littermates. The insets on the right panel are high-magnification images of the indicated portions (squares) of the images on the left. The morphology of mitochondria in Pgam2 mice was normal. Nucl.: nucleus. The bar represents 1 µm. The density (number per 100 µm2) and the size (μm2) of mitochondria within a cardiomyocyte were observed by electron microscopy (lower panels). Values are the mean ± SEM (n = 3 for each groups). (B) TBARS as a marker of oxidative stress. Thiobarbituric acid reactive substances (TBARS) levels were normal in the heart tissue of Pgam2 mice. Values are the mean ± SEM. NTg mice: n = 7; Pgam2 mice: n = 8. (TIF) [file pone.0072173.s002.tif]
