# Supplementary material for: Persistent Overexpression of Phosphoglycerate Mutase, a Glycolytic Enzyme, Modifies Energy Metabolism and Reduces Stress Resistance of Heart in Mice
Source: PLoS One. 2013 Aug 12;8(8):e72173. doi: 10.1371/journal.pone.0072173 (PMC3741204; doi:10.1371/journal.pone.0072173)
Supplement: Table S1 — Primer sequences used for real-time quantitative RT-PCR. (DOC) [file pone.0072173.s003.doc]

**Table S1.** Primer sequences used for real-time quantitative RT-PCR

| Gene | Forward primer | Reverse primer |
| --- | --- | --- |
| *18S rRNA* | CGGCTACCACATCCAAGGAA | GCTGGAATTACCGCGGCT |
| *Hexokinase 1* | ACGTTTTCCTTCCCGTGCCGA | AGCGTCATAGTCCCCTCGCTTCT |
| *Hexokinase 2* | CCAGACGAAACTGGATGAGA | CAGCCACAATGTCAATGTCA |
| *Glucokinase* | AGAAGGAAAAGGTAGAGCAGA | TCTTTACACTGGCCTCCTGA |
| *Pfkfb1* | TCAGCTCGGGGCAAGCAGTATG | AGTGTCCAGGGCTTCCTCGGG |
| *Pfkfb2* | CCGATACCCTGGTGGGGAGTCCTA | GCGCATGACAGCCTGGTGAGA |
| *Pfk1* | ATGGAGTGCGTGCAGGTGACCA | TCATCACGGCCACTGTGTGCAA |
| *PK* | GGATACAAAGGGACCTGAGA | GCAGATGTTCTTGTAGTCCA |
| *Hif-1α* | GATGGCTCCCTTTTTCAAGCA | CCCAGCAGTCTGCATGCTA |
| *PGC1α* | TTCTGGGTGGATTGAAGTGGTG | TGTCAGTGCATCAAATGAGGGC |
| *PPARα* | GAGAATCCACGAAGCCTACC | AATCGGACCTCTGCCTCTTT |
| *PPARδ* | CCGCCGGACAATCCGCATGA | AGCGGATAGCGTTGTGCGACA |
| *ERRα* | CAAGAGCATCCCAGGCTT | GCACTTCCATCCACACACTC |
| *NRF-1* | GAACTGCCAACCACAGTCAC | TTTGTTCCACCTCTCCATCA |
| *Tfam* | CCAAAAAGACCTCGTTCAGC | ATGTCTCCGGATCGTTTCAC |
| *CD36/FAT* | GGCCAAGCTATTGCGACAT | CAGATCCGAACACAGCGTAGA |
| *CPT-1b* | TGCCTTTACATCGTCTCCAA | AGACCCCGTAGCCATCATC |
| *MCAD* | GATCGCAATGGGTGCTTTTGATAGAA | AGCTGATTGGCAATGTCTCCAGCAAA |
| *IDH3α* | CCACAACACAAAACAGGTGA | GTCCTTGAATTGCTGTGACA |
| *OgDh* | CCCTGGGGATTTTGGATGCT | TGTAGGCCATAGAACCCTCCT |
| *SCS* | CAGGGAACGACGAGGCTAAT | GTACCGGACTTGGACACGAT |
| *ND4* | catcactcctattctgcctagcaa | tcctcgggccatgattatagtac |
| *α-S9* | cccgggccagcttacct | gctgcactgctttcctgataga |
| *SDHB* | cgctgccacaccatcatg | tttccgcaatcgctttcc |
| *Fe-S* | gctgggcgcacactttgt | cactggccttgcaggaagaa |
| *Cyt-b* | gccaccttgacccgattct | ttgctagggccgcgataat |
| *Cyt-c* | ggaggcaagcataagactgg | tccatcagggtatcctctcc |
| *COX5a* | GGGTCACACGAGACAGATGA | GGAACCAGATCATAGCCAACA |
| *COX7a1* | GTCTCCCAGGCTCTGGTCCG | CTGTACAGGACGTTGTCCATTC |
| *UCP2* | CTCTGCCTTGGGCCAGTACCA | GTTCCAGGATCCCAAGCGGAGA |
| *UCP3* | ACTCCAGCGTCGCCATCAGGATTCT | TAAACAGGTGAGACTCCAGCAACTT |
| *Mn-SOD* | gcgcagatcatgcagctgca | caggctgaagagcgacctga |
