# Supplementary material for: Persistent Overexpression of Phosphoglycerate Mutase, a Glycolytic Enzyme, Modifies Energy Metabolism and Reduces Stress Resistance of Heart in Mice
Source: PLoS One. 2013 Aug 12;8(8):e72173. doi: 10.1371/journal.pone.0072173 (PMC3741204; doi:10.1371/journal.pone.0072173)
Supplement: Table S2 — Myocardial uptake of 18FDG and 125I-9MPA. (DOC) [file pone.0072173.s004.doc]

**Table S2.** Myocardial uptake of ^18^FDG and ^125^I-9MPA.

|  | Number of samples | **^18^FDG** | | **^125^I-9MPA** | |
| --- | --- | --- | --- | --- | --- |
|  |  | CPM | SUV | CPM | SUV |
| Hearts with RI |  |  |  |  |  |
| NTg | 13 | 262888 ± 34414 | 283 ± 36 | 113429 ± 7510 | 473 ± 26 |
| Pgam2 | 18 | 349711 ± 37166 | 246 ± 32 | 111085 ± 4514 | 542 ± 28 |
| Hearts without RI | 6 | N.D. | N.A. | 37 ± 3 | N.A. |
| Empty tubes | 6 | N.D. | N.A. | 26 ± 4 | N.A. |

Radioisotope (RI) counts per minute (CPM) of each sample were corrected by the average counts of the RI in hearts without RI and those of empty tubes. SUV: standard uptake value. Values are the mean ± SEM. N.D.: not determined; N.A.: not available.
