# Supplementary material for: Persistent Overexpression of Phosphoglycerate Mutase, a Glycolytic Enzyme, Modifies Energy Metabolism and Reduces Stress Resistance of Heart in Mice
Source: PLoS One. 2013 Aug 12;8(8):e72173. doi: 10.1371/journal.pone.0072173 (PMC3741204; doi:10.1371/journal.pone.0072173)
Supplement: Table S3 — Concentrations of metabolites in the heart identified by metabolomic analysis. (DOC) [file pone.0072173.s005.doc]

**Table S3.** Concentrations of metabolites in the heart identified by metabolomic analysis.

| KEGG ID |  | NTg | | | Pgam2 | | |  |
| --- | --- | --- | --- | --- | --- | --- | --- | --- |
|  |  | (n = 5) | | | (n= 5) | | |  |
|  | ***Glycolysis and intermediates*** |  |  |  |  |  |  |  |
| C00160 | Glycolate | 57.30 | ± | 6.09 | 60.22 | ± | 7.22 |  |
| C00103 | Glucose-1-phosphate | 91.56 | ± | 8.38 | 85.17 | ± | 6.00 |  |
| C00092 | Glucose-6-phosphate | 743.57 | ± | 107.63 | 658.36 | ± | 95.80 |  |
| C00085 | Fructose-6-phosphate | 115.46 | ± | 15.69 | 92.41 | ± | 11.35 |  |
| C00354 | Fructose 1,6-bisphosphate | 487.44 | ± | 43.44 | 472.23 | ± | 39.00 |  |
| C00197 | 3-Phosphoglycerate | 54.50 | ± | 2.91 | 121.71 | ± | 9.96 | * |
| C01159 | 2,3-Diphosphoglycerate | 63.05 | ± | 6.34 | 34.69 | ± | 3.31 | * |
| C00631 | 2-Phosphoglycerate | 4.41 | ± | 0.35 | 12.84 | ± | 0.90 | * |
| C00074 | Phosphoenolpyruvate (PEP) | 3.19 | ± | 0.21 | 7.29 | ± | 0.42 | * |
| C00186 | Lactate | 8615.59 | ± | 358.66 | 8053.05 | ± | 281.70 |  |
| C00258 | Glycerate | 8.84 | ± | 1.69 | 54.53 | ± | 3.30 | * |
|  |  |  |  |  |  |  |  |  |
|  | ***TCA cycle and intermediates*** |  |  |  |  |  |  |  |
| C00024 | Acetyl CoA | 17.07 | ± | 0.90 | 19.66 | ± | 0.40 | * |
| C00158 | Citrate | 461.40 | ± | 40.15 | 579.37 | ± | 37.80 |  |
| C00417 | cis-Aconitate | 12.89 | ± | 1.14 | 17.50 | ± | 1.55 | * |
| C00311 | Isocitrate | 38.63 | ± | 1.95 | 47.21 | ± | 4.24 |  |
| C00647 | Pyridoxamine 5'-phosphate | 29.27 | ± | 0.60 | 31.34 | ± | 0.46 | * |
| C02630 | 2-Hydroxyglutarate | 35.95 | ± | 3.81 | 18.73 | ± | 2.47 | * |
| C00042 | Succinate | 761.22 | ± | 22.18 | 719.69 | ± | 27.56 |  |
| C00122 | Fumarate | 94.26 | ± | 5.70 | 71.14 | ± | 2.77 | * |
| C00711 | Malate | 518.49 | ± | 34.07 | 429.33 | ± | 19.97 |  |
| C00083 | Malonyl CoA | 4.00 | ± | 0.44 | 4.55 | ± | 0.44 |  |
| C00383 | Malonate | 2.00 | ± | 0.31 | 3.00 | ± | 0.37 |  |
|  |  |  |  |  |  |  |  |  |
|  | ***Amino acids and intermediates*** |  |  |  |  |  |  |  |
| C00065 | Serine | 427.71 | ± | 28.89 | 232.13 | ± | 9.31 | * |
| C00263 | Homoserine | 4.95 | ± | 0.29 | 4.44 | ± | 0.32 |  |
| C00037 | Glycine | 543.12 | ± | 18.64 | 511.14 | ± | 21.58 |  |
| C00719 | Betaine | 86.25 | ± | 9.99 | 59.96 | ± | 5.09 | * |
| C00114 | Choline | 54.85 | ± | 1.84 | 58.18 | ± | 3.64 |  |
| C00334 | GABA | 7.16 | ± | 0.80 | 9.96 | ± | 1.01 |  |
| C00073 | Methionine | 76.30 | ± | 4.42 | 65.26 | ± | 3.85 |  |
| C00127 | GSSG (oxidized-form glutathione) | 279.03 | ± | 12.38 | 251.33 | ± | 7.50 |  |
| C00051 | GSH (reduced-form glutathione) | 630.55 | ± | 35.60 | 448.95 | ± | 8.45 | * |
|  | Total glutathione (GSSG+GSH) | 909.58 | ± | 31.03 | 700.28 | ± | 15.03 | * |
| C00245 | Taurine | 6149.30 | ± | 515.10 | 5708.18 | ± | 563.01 |  |
| C00519 | Hypotaurine | 285.89 | ± | 13.61 | 271.61 | ± | 18.60 |  |
| C05123 | Isethionate | 36.48 | ± | 2.63 | 50.24 | ± | 3.73 | * |
| C00041 | Alanine | 1951.11 | ± | 95.33 | 1723.70 | ± | 94.25 |  |
| C00049 | Aspartate | 1199.97 | ± | 232.30 | 595.62 | ± | 49.26 | * |
| C03794 | Adenylosuccinate | 26.40 | ± | 2.93 | 16.99 | ± | 1.46 | * |
| C00047 | Lysine | 512.63 | ± | 12.42 | 548.75 | ± | 38.77 |  |
| C00318 | Carnitine | 404.31 | ± | 27.11 | 348.82 | ± | 17.47 |  |
| C00099 | beta Alanine | 23.73 | ± | 3.09 | 16.81 | ± | 2.73 |  |
| C00135 | Histidine | 257.48 | ± | 12.44 | 289.99 | ± | 6.52 | * |
| C00388 | Histamine | 6.14 | ± | 0.41 | 5.97 | ± | 0.29 |  |
| C05127 | 1-Methylhistamine | 0.63 | ± | 0.06 | 0.61 | ± | 0.07 |  |
| C00386 | Carnosine | 27.71 | ± | 2.85 | 71.18 | ± | 6.10 | * |
| C01262 | Anserine | 7.58 | ± | 0.74 | 20.21 | ± | 1.70 | * |
| C00025 | Glutamate | 3897.49 | ± | 132.17 | 4244.17 | ± | 113.87 |  |
| C00148 | Proline | 92.73 | ± | 5.00 | 76.96 | ± | 3.72 | * |
| C01015 | Hydroxyproline | 66.24 | ± | 5.17 | 40.63 | ± | 3.38 | * |
| C00077 | Ornithine | 11.18 | ± | 0.56 | 10.08 | ± | 0.68 |  |
| C00327 | Citrulline | 188.82 | ± | 6.67 | 188.03 | ± | 7.65 |  |
| C03406 | Argininosuccinate | 4.89 | ± | 0.25 | 3.63 | ± | 0.30 | * |
| C00062 | Arginine | 242.61 | ± | 6.30 | 254.22 | ± | 10.10 |  |
| C00300 | Creatine | 6444.53 | ± | 265.19 | 6059.07 | ± | 142.41 |  |
| C00791 | Creatinine | 36.87 | ± | 3.02 | 31.46 | ± | 1.04 |  |
| C00134 | Putrescine(1,4-Butanediamine) | 3.96 | ± | 0.28 | 3.12 | ± | 0.18 | * |
| C00315 | Spermidine | 33.13 | ± | 1.67 | 27.23 | ± | 1.23 | * |
| C00750 | Spermine | 5.14 | ± | 0.81 | 5.20 | ± | 0.57 |  |
| C00123 | Leucine | 107.70 | ± | 6.00 | 94.18 | ± | 5.00 |  |
| C00407 | Isoleucine | 62.67 | ± | 5.42 | 52.80 | ± | 3.69 |  |
| C00183 | Valine | 143.90 | ± | 9.81 | 127.51 | ± | 6.78 |  |
| C00079 | Phenylalanine | 64.63 | ± | 1.72 | 62.18 | ± | 2.59 |  |
| C00082 | Tyrosine | 90.31 | ± | 7.71 | 94.23 | ± | 6.35 |  |
| C00152 | Asparagine | 164.68 | ± | 4.29 | 150.20 | ± | 8.82 |  |
| C00064 | Glutamine | 4754.46 | ± | 192.58 | 5065.96 | ± | 112.85 |  |
| C00188 | Threonine | 349.37 | ± | 12.68 | 324.90 | ± | 11.86 |  |
| C00078 | Tryptophan | 23.69 | ± | 1.10 | 25.33 | ± | 0.57 |  |
| C00262 | Hypoxanthine | 16.56 | ± | 0.81 | 15.07 | ± | 1.13 |  |
| C00019 | S-Adenosylmethionine | 36.57 | ± | 0.95 | 36.79 | ± | 1.22 |  |
| - | Ophthalmate | 0.87 | ± | 0.09 | 0.82 | ± | 0.09 |  |
| C02356 | 2-Aminobutyrate (2AB) | 5.83 | ± | 0.50 | 4.24 | ± | 0.25 | * |
|  |  |  |  |  |  |  |  |  |
| C00003 | NAD^+^ | 564.30 | ± | 26.46 | 594.49 | ± | 26.03 |  |
| C00004 | NADH | 272.11 | ± | 13.95 | 263.20 | ± | 19.70 |  |
| C00005 | NADPH | 72.81 | ± | 5.88 | 93.47 | ± | 5.47 | * |
| C00006 | NADP^+^ | 43.06 | ± | 2.94 | 46.83 | ± | 3.07 |  |

Values are expressed in nmol/g, and are presented as the mean ± SEM. NTg: non-transgenic mice; Pgam2: phosphoglycerate mutase 2 transgenic mice. *p < 0.05 versus NTg mice.
